# Gliadin Peptides Induce Tissue Transglutaminase Activation and ER-Stress through Ca^2+^ Mobilization in Caco-2 Cells

**DOI:** 10.1371/journal.pone.0045209

**Published:** 2012-09-25

**Authors:** Ivana Caputo, Agnese Secondo, Marilena Lepretti, Gaetana Paolella, Salvatore Auricchio, Maria Vittoria Barone, Carla Esposito

**Affiliations:** 1 Department of Chemistry and Biology, University of Salerno, Fisciano, Salerno, Italy; 2 European Laboratory for the Investigation of Food-Induced Diseases (ELFID), University Federico II, Naples, Italy; 3 Department of Neurosciences, University Federico II, Naples, Italy; 4 Department of Pediatrics, University Federico II, Naples, Italy; Consejo Superior de Investigaciones Cientificas, Spain

## Abstract

**Background:**

Celiac disease (CD) is an intestinal inflammatory condition that develops in genetically susceptible individuals after exposure to dietary wheat gliadin. The role of post-translational modifications of gliadin catalyzed by tissue transglutaminase (tTG) seems to play a crucial role in CD. However, it remains to be established how and where tTG is activated *in vivo*. We have investigated whether gliadin peptides modulate intracellular Ca^2+^ homeostasis and tTG activity.

**Methods/Principal Findings:**

We studied Ca^2+^ homeostasis in Caco-2 cells by single cell microfluorimetry. Under our conditions, A-gliadin peptides 31–43 and 57–68 rapidly mobilized Ca^2+^ from intracellular stores. Specifically, peptide 31–43 mobilized Ca^2+^ from the endoplasmic reticulum (ER) and mitochondria, whereas peptide 57–68 mobilized Ca^2+^ only from mitochondria. We also found that gliadin peptide-induced Ca^2+^ mobilization activates the enzymatic function of intracellular tTG as revealed by *in situ* tTG activity using the tTG substrate pentylamine-biotin. Moreover, we demonstrate that peptide 31–43, but not peptide 57–68, induces an increase of tTG expression. Finally, we monitored the expression of glucose-regulated protein-78 and of CCAAT/enhancer binding protein-homologous protein, which are two biochemical markers of ER-stress, by real-time RT-PCR and western blot. We found that chronic administration of peptide 31–43, but not of peptide 57–68, induces the expression of both genes.

**Conclusions:**

By inducing Ca^2+^ mobilization from the ER, peptide 31–43 could promote an ER-stress pathway that may be relevant in CD pathogenesis. Furthermore, peptides 31–43 and 57–68, by activating intracellular tTG, could alter inflammatory key regulators, and induce deamidation of immunogenic peptides and gliadin–tTG crosslinking in enterocytes and specialized antigen-presenting cells.

## Introduction

Celiac disease (CD) is a complex inflammatory condition of the proximal small intestine caused by a specific response to peptides derived from ingested gliadin [1]. Immunotoxic gliadin peptides initiate a deleterious adaptive and innate immune response in the intestinal epithelium of CD patients. A-gliadin peptide 31-43/49 (p31–43) is the prototype of peptides that modulate the innate response [2], whereas peptide 57–68 (p57–68), which binds to HLA-DQ2/8 molecules, is one of the dominant epitopes recognized by T cells isolated from the intestine of CD patients [3]. However, the innate and adaptive immune systems may respond synergistically to gliadin peptides [1]. The role of post-translational modifications of gliadin peptides catalyzed by tissue transglutaminase (tTG) is thought to play a crucial role in CD [4], [5].

Tissue TG is a Ca^2+^-dependent enzyme that catalyzes the formation of isopeptide linkages between the γ-carboxamide group of protein-bound glutamine residues and the ε-amino group of protein-bound lysine residues [6]. Glutamine residues can be deamidated to glutamic acid as a side-reaction in the absence of suitable amines or at low pH. Furthermore, tTG also binds and GTP; hence the enzyme can function as a cell signal transducer in association with the α_1β_-adrenoreceptor [7]. Tissue TG is predominantly an intracellular protein localized in the cytosol, mitochondria, nucleus, and cell membrane compartments [6], but it is also secreted extracellularly even though it lacks a signal leader peptide. Recently, Zemskov *et al.* described secretion of tTG that involves phospholipid-dependent delivery into recycling endosomes [8].

Various functions have been ascribed to tTG in both the intra- and extracellular environment: in fact, it plays a role in matrix stabilization, cell adhesion and migration, and in cell death and survival [6], [9], [10]. The catalytic activity of tTG is implicated in the pathogenesis of several human diseases, including CD [10]. In celiac patients, tTG deamidates specific gliadin glutamines, thus generating a series of gliadin peptides that bind to HLA-DQ2 and DQ8 molecules with high affinity. The resulting HLA-DQ2 (DQ8)-gliadin peptide interaction triggers the proinflammatory T cell response [1]. Moreover, in accordance with the upregulation of tTG in intestinal inflamed sites, tTG may generate additional antigenic epitopes by cross-linking gliadin peptides to itself or to other cellular proteins. Gliadin-tTG complexes may elicit an immune response to tTG by stimulating normally silent autoreactive B-cells [11], [12]. In fact, active CD is associated with serum antibodies against tTG.

The exact location at which deamidation of immunogenic gliadin peptides and formation of gliadin–tTG complexes take place is not clear. Although little is known about the processing of gliadin peptides, there is evidence that they enter enterocytes [13], [14]. However, do tTG-induced gliadin modifications in CD patients occur in enterocytes and/or in other antigen-presenting cells, or in the extracellular matrix? It has been demonstrated that extracellular tTG is inactive in the intestinal mucosa in the resting state and it is only transiently activated after some inflammatory stimuli and tissue injury [15]. Moreover, under normal conditions, tTG in the intracellular environment is a latent protein due to a low Ca^2+^ concentration and inhibition by GTP/GDP. However, under extreme conditions of cell stress or trauma, and after disturbance or loss of Ca^2+^ homeostasis, tTG may be activated and cause cross-linking of intracellular proteins, as observed during apoptosis or necrosis [16], [17]. It has been reported that p31–43 causes increased production of reactive oxygen species, which leads to tTG overexpression and activation in intestinal epithelial cells; active tTG then induces ubiquitination and degradation of the peroxisome proliferator-activated receptor (PPAR)γ [18], thus contributing to the inflammatory response. Although Ca^2+^ is a prerequisite for activation of tTG, and for the production of reactive oxygen species, the role of this important cellular ionic mediator in the pathogenesis of CD remains unknown and unexplored.

We carried out the present study to determine the effect of gliadin peptides on Ca^2+^ homeostasis in the attempt to gain additional insights into the possible role exerted by gliadin peptides in the molecular mechanisms of tTG activation. Here we demonstrate that dietary wheat gliadin is able to regulate intracellular Ca^2+^ homeostasis. In fact, gliadin peptides rapidly mobilize Ca^2+^ ions from intracellular stores in a cell model of intestinal epithelial cells. However, p31–43 at a low concentration mobilizes calcium from the endoplasmic reticulum (ER), whereas both p31–43 and a higher concentration of p57–68 mobilize Ca^2+^ from mitochondria. We also show that Ca^2+^ ions released from intracellular stores as a consequence of gliadin peptide stimulation, are able to activate cytosolic and nuclear tTG. Finally, we found that p31–43, but not p57–68, increases the expression of tTG. It also increased the expression of glucose-regulated protein (GRP)-78 and of CCAAT/enhancer binding protein-homologous protein (CHOP) thereby implicating the ER-stress pathway in CD mucosal damage.

## Methods

### Peptides

Synthetic peptides were from Inbios (Naples, Italy). The sequences of p31–43 and p57–68 from A-gliadin were LGQQQPFPPQQPY and QLQPFPQPQLPY, respectively. In some experiments, we used N-terminal-biotin-labeled p31–43 and p57–68. Three synthetic peptide were used as irrelevant control peptides: QQPQDAVQPF from durum wheat (pCTR) [19]; PLIRPLLARPAK, which represents the 537–548 region of the human thyroid peroxidase (pHTP) [2]; LPQFEEIRNLALQTLPAM, which represents the C-terminal sequence of A-gliadin (p229–246) [20].

**Figure 1:**
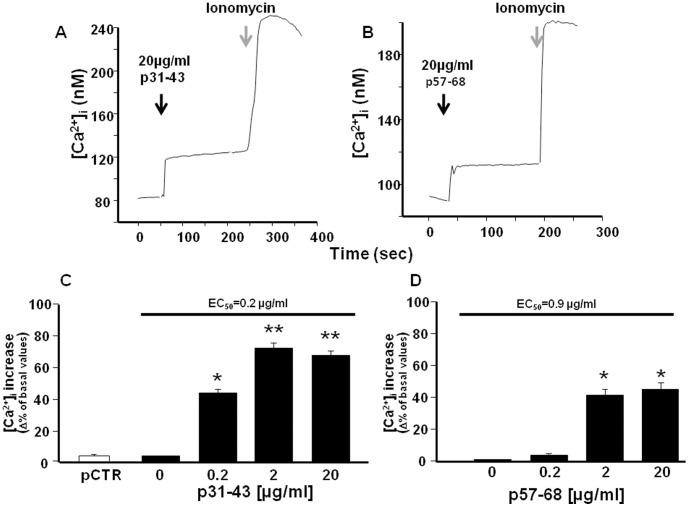
Effect of p31–43 and p57–68 on [Ca^2+^]_i_ in Caco-2 cells. (**A**) and (**B**) Single-cell traces representative of the effect of 20 µg/ml p31–43 and p57–68, respectively, on [Ca^2+^]_i_. Starting time of perfusion is indicated by the arrows. Ionomycin (1µM) was added as control (grey arrows) at the end of the experiment. (**C**) and (**D**) Dose-dependent effect of p31–43 and p57–68, respectively, on [Ca^2+^]_i_ increase. PCTR (20 µg/ml) served as control. From 40–65 cells were monitored in each experiment. Each bar represents the mean ± SEM of data obtained in three independent experimental sessions. *p<0.05 versus its respective control (basal values); **p<0.05 versus previous concentrations and control.

### Cell Culture

Caco-2 cells were obtained from Interlab Cell Line Collection (Centro di Biotecnologie Avanzate, Genoa, Italy). Caco-2 cells were cultured in 100×10-mm Petri dishes containing Dulbecco’s modified Eagle’s medium supplemented with 10% (v/v) fetal bovine serum, 1% (v/v) non-essential amino acids, 0.2 mM L-glutamine, 50 units/ml penicillin and 50 µg/ml streptomycin (Invitrogen SRL, Milan, Italy). Cells were maintained at 37°C in a 5% CO_2_, 95% air-humidified atmosphere and passaged twice a week. Treatments were generally performed after 72–96 h after seeding.

**Figure 2:**
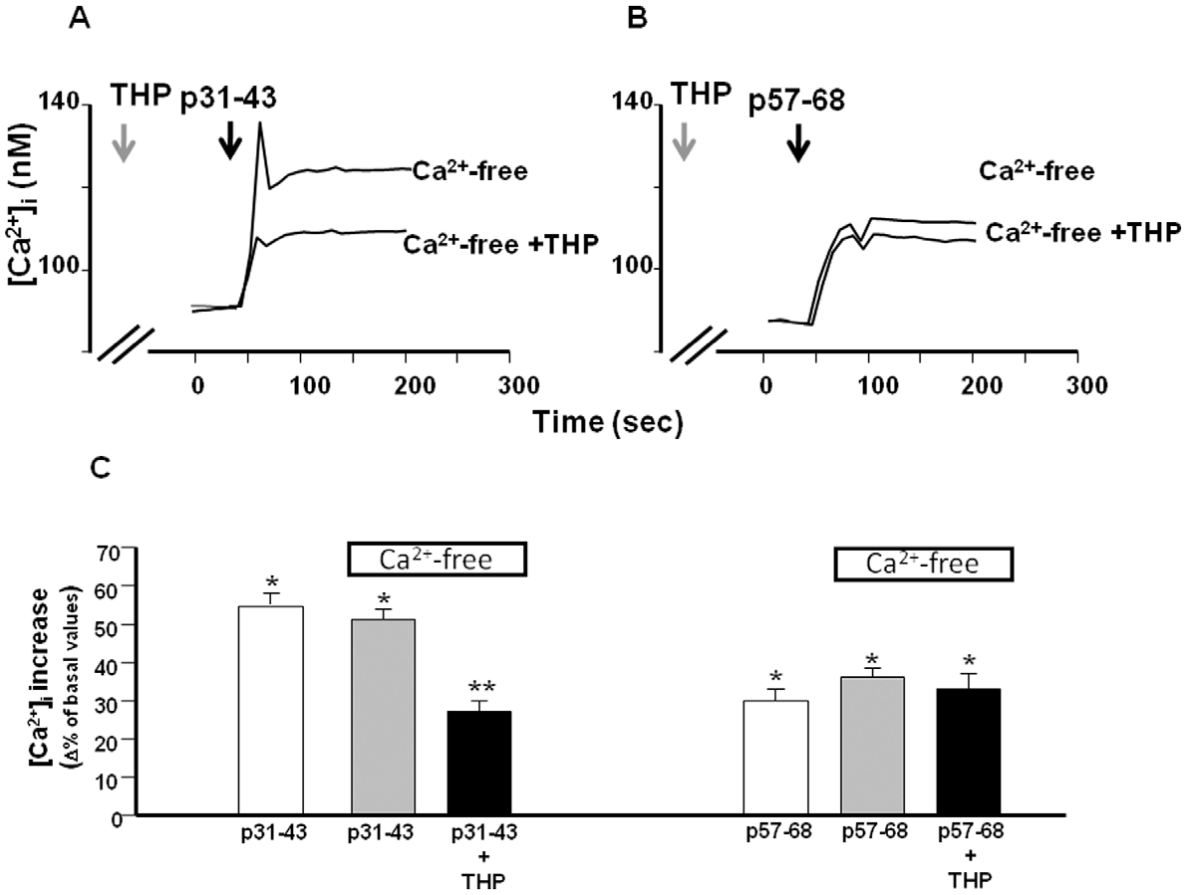
Effect of Ca^2+^-free and THP on [Ca^2+^]_i_ increase induced by gliadin peptides in Caco-2 cells. (**A**) and (**B**) Superimposed single-cell traces representative for the effect of 20 µg/ml p31–43 and p57–68, respectively, in a Ca^2+^-free buffer, or in a Ca^2+^-free buffer plus 1 µM THP, on [Ca^2+^]_i_. Before peptide perfusion (black arrows), cells were preincubated with THP for 10 min (grey arrows), to deplete ER. (**C**) Quantification of the effect of the treatments reported in (A) and (B) on [Ca^2+^]_i_. Each bar represents the mean ±SEM of data obtained in three independent experimental sessions. *p<0.05 versus its respective control; **p<0.05 versus peptide alone.

**Figure 3:**
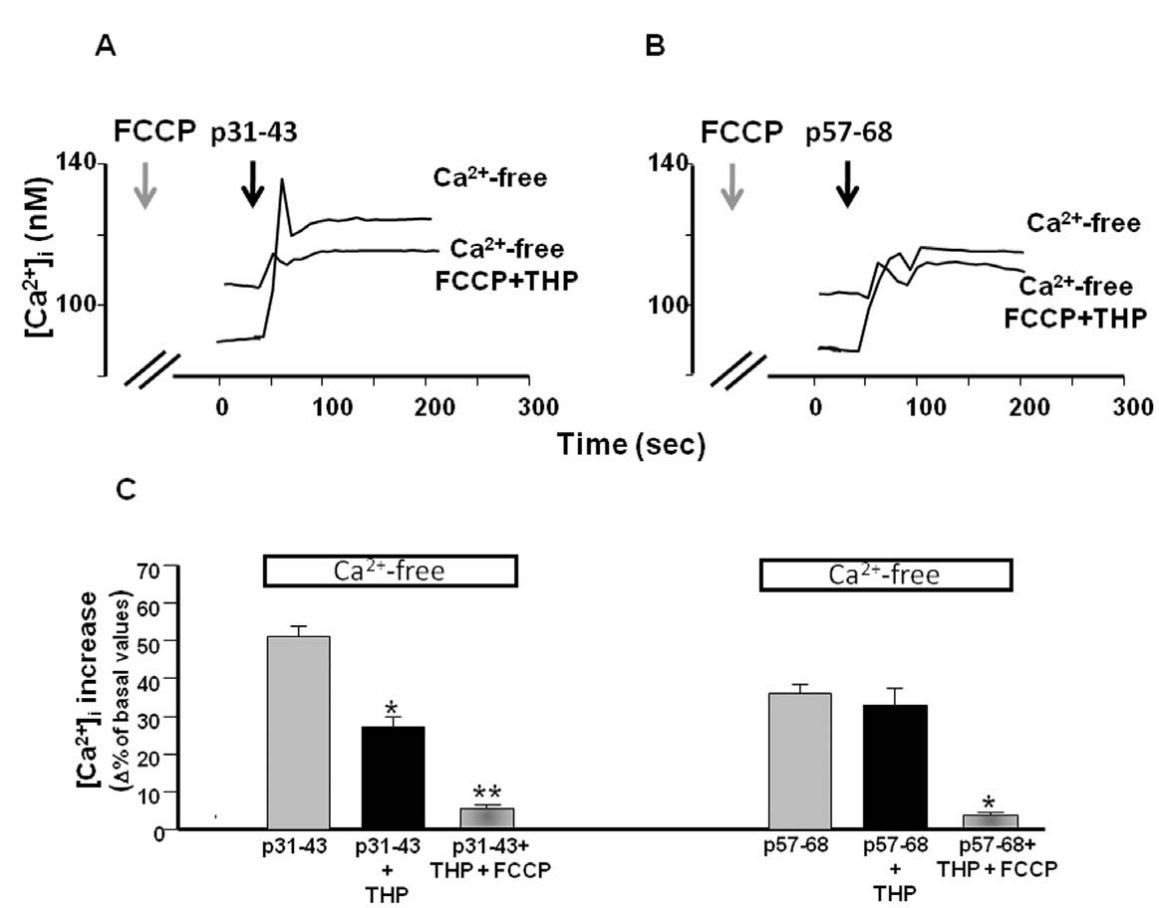
Effect of FCCP on [Ca^2+^]_i_ increase induced by gliadin peptides in Caco-2 cells. (**A**) and (**B**) Superimposed single-cell traces representative of the effect of 20 µg/ml p31–43 and p57–68, respectively, in a Ca^2+^-free buffer, and in a Ca^2+^-free buffer plus 1µM THP and 300 nM FCCP, on [Ca^2+^]_i_. Before peptide perfusion (black arrows), cells were preincubated with FCCP and THP for 10 min (grey arrows). (**C**) Quantification of the effect of the treatments reported in (A) and (B) on [Ca^2+^]_i_. Each bar represents the mean ± SEM of data obtained in three independent experimental sessions. *p<0.05 versus peptide alone; **p<0.05 versus peptide plus THP.

### Intracellular Ca^2+^ Concentration Measurement

Intracellular Ca^2+^ concentration ([Ca^2+^]_i_) was measured in Caco-2 cells by single cell computer-assisted videoimaging using the Ca^2+^ indicator Fura-2 acetoxymethyl ester, as previously described [21], [22]. Gliadin peptides were loaded at concentrations between 0.2 and 20 µg/ml for 30 min at 37°C in normal Krebs solution containing the following (in mM): 5.5 KCl, 160 NaCl, 1.2 MgCl_2_, 1.5 CaCl_2_, 10 glucose, and 10 Hepes-NaOH, pH 7.4. To selectively deplete intracellular Ca^2+^ stores, experiments were performed in the presence of thapsigargin (THP) (1 µM), an irreversible and selective inhibitor of the sarco(endo)plasmic reticulum Ca^2+^ ATPase, and in the presence of the mitochondrial uncoupler carbonylcyanide-p-trifluoromethoxyphenylhydrazone (FCCP) (300 nm).

**Figure 4:**
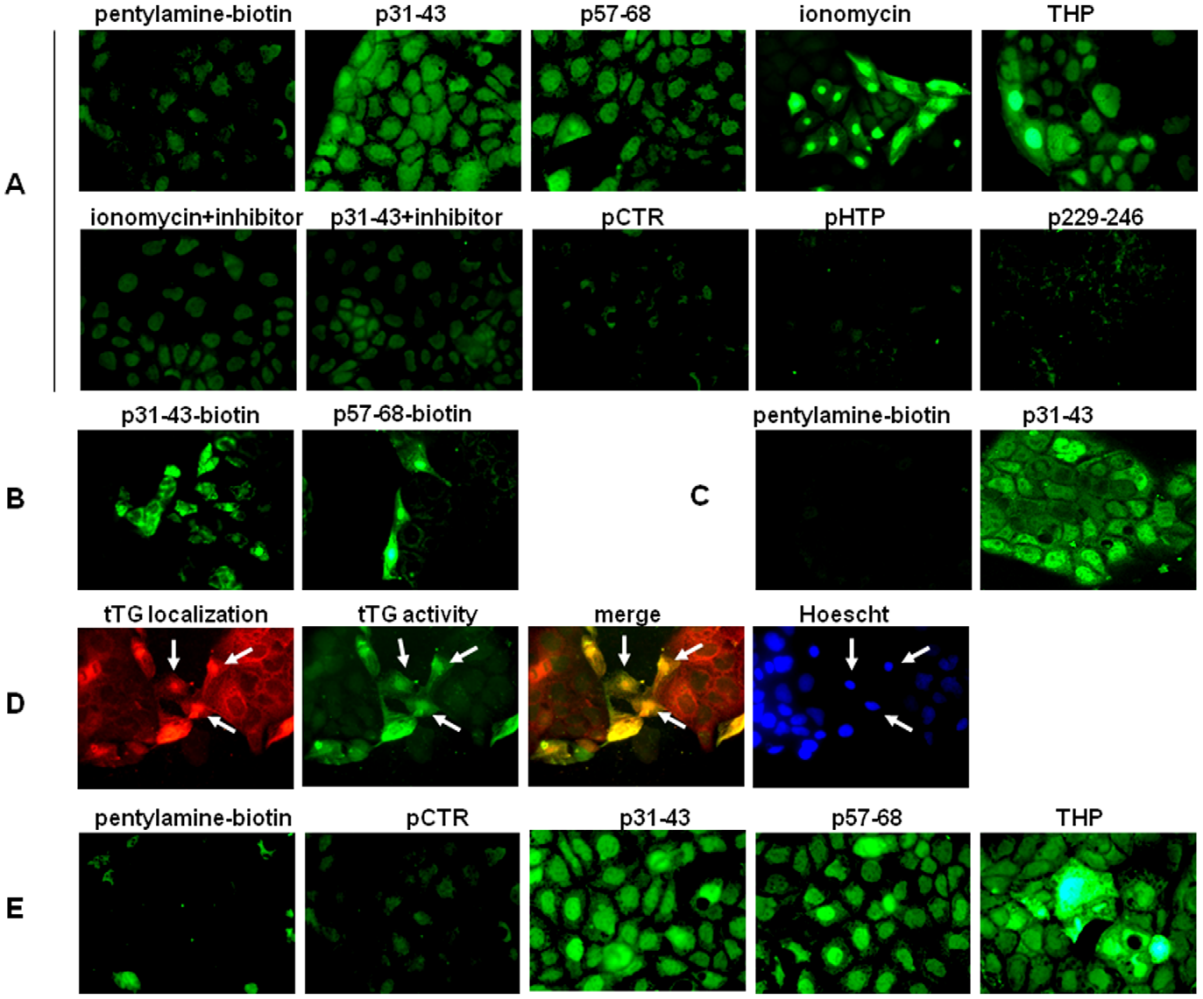
Microscopic visualization of tTG transamidating activity in Caco-2 cells. (**A**) Microscopic visualization of pentylamine-biotin incorporation *in situ*, ×40, in the presence of a complete medium. Peptides were used at 20 µg/ml, ionomycin at 10 µM, and THP at 1µM. Where indicated, the tTG inhibitor cystamin (200 µM) was added 5 min before treatment. (**B**) Microscopic visualization of p31–43-biotin and p57–68-biotin incorporation *in situ*, x40, in the presence of ionomycin. (**C**) Confocal images of pentylamine-biotin incorporation *in situ*, x63 (LSM 510 Zeiss microscope), in the absence or presence of p31–43 20 µg/ml. (**D**) tTG localization (red) in ionomycin-treated cells and superimposition (merge) with intracellular tTG activity (green). Nuclei are in blue (Hoescht staining). Arrows indicate nuclei in which tTG is increased (**E**) Microscopic visualization of pentylamine-biotin incorporation *in situ*, ×40, in the presence of a Ca^2+^-free medium.

**Figure 5:**
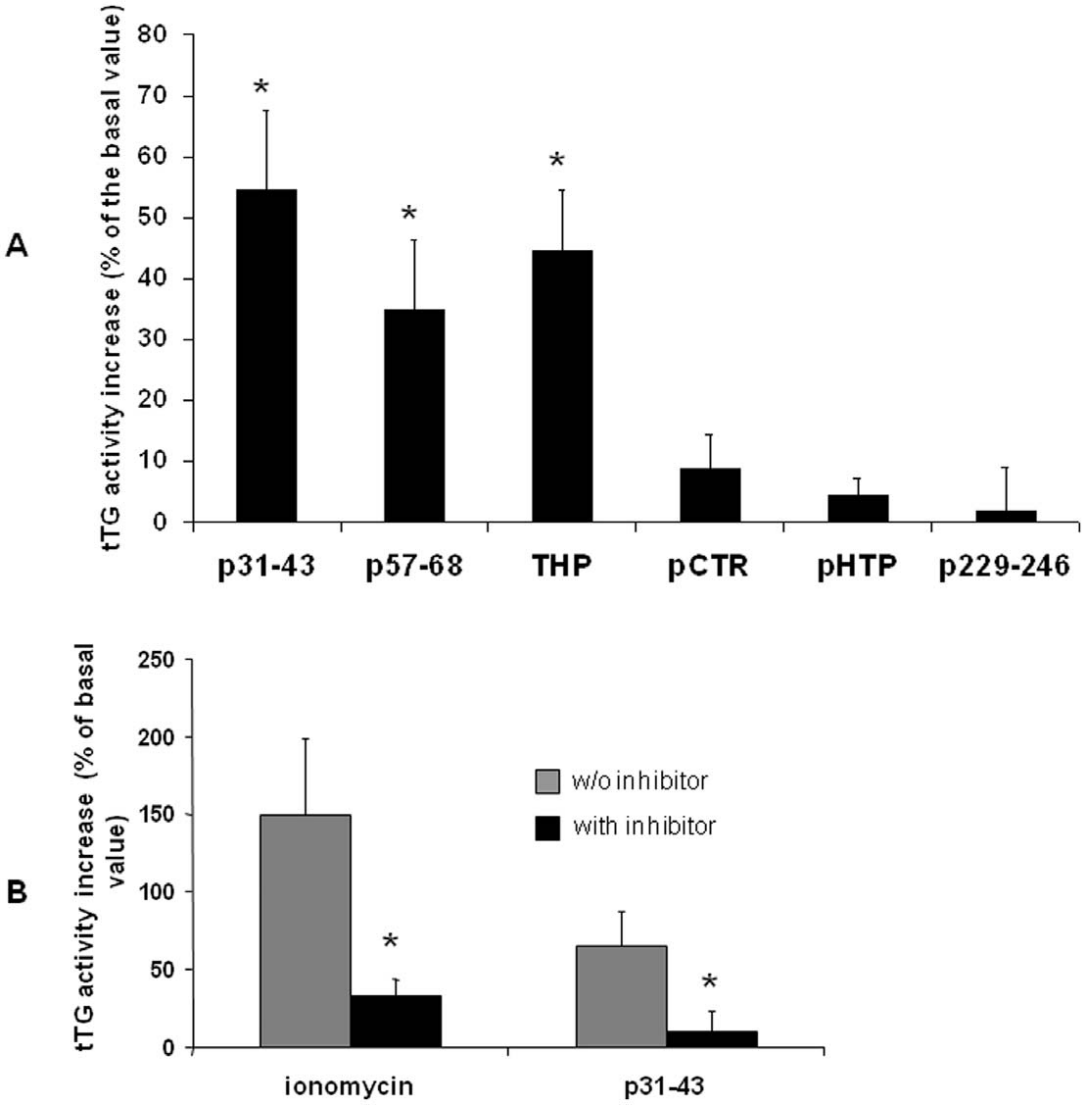
Quantification of tTG activity by the microplate assay. (**A**) The microplate assay was performed on 25 µg of cell lysates obtained after treatment. Values are the means ± SD of at least 3 independent experiments in triplicate. *p<0.05 *versus* tTG basal activity. (**F**) Inhibition by cystamin of tTG activity induced by ionomycin and p31–43. Values are the means ± SD of three independent experiments in triplicate. *p<0.05 *versus* the respective activity in the absence of the inhibitor.

### In situ tTG Assay and Quantification of tTG Transamidating Activity

*In situ* tTG activity was visualized by using the tTG substrate pentylamine-biotin (Euroclone, Milan, Italy) as reported elsewhere [22]. Caco-2 cells were treated for 30 min with 10 µM ionomycin (Sigma-Aldrich, Milan, Italy), or with different amounts of gliadin peptides, or with 1 µM THP, in the presence of 0.5 mM pentylamine-biotin, before being processed for microscopy observation. To visualize biotin-labeled p31–43 and p57–68 in the cells, peptides needed to be used at a concentration of 0.2 mM. Tissue TG localization in permeabilized cells was revealed by incubating cells with the mouse anti-tTG antibody, clone CUB7402 (Bioptica, Milan, Italy), diluted 1∶200, and a secondary TRITC-conjugated antibody (1∶200). Pentylamine-biotin was also used to quantify the tTG transamidating activity by a microplate assay [22]. Briefly, after 30 min of treatment with 20 µg/ml gliadin peptides, or with 1 µM THP, cells were lysed and proteins (25 µg) were coated into the wells of a 96-well plate. The wells were blocked with 10% bovine serum albumin then incubated with 1∶5000 peroxidase-conjugated streptavidin (Euroclone) in 5% bovine serum albumin. To reveal peroxidase activity, 3,3′,5,5′-tetramethylbenzidine (Sigma-Aldrich, Milan, Italy) was added to each well and, after stopping the reaction with H_2_SO_4_, absorbances were read at 450 nm.

**Figure 6:**
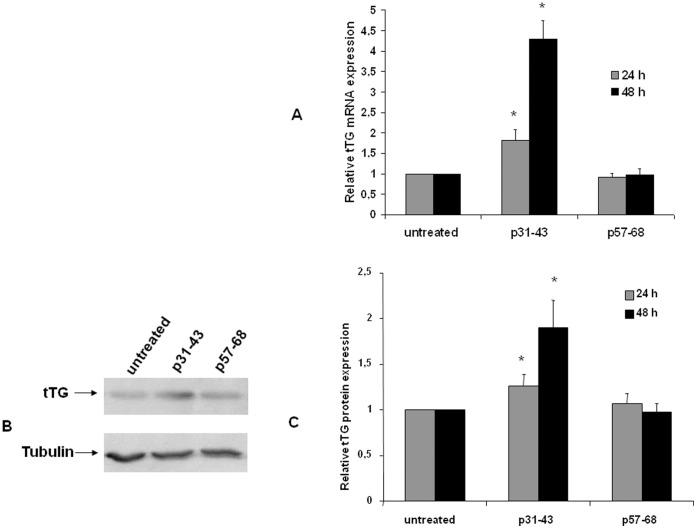
Analysis of p31–43 induced tTG expression in Caco-2 cells. (**A**) Quantification of tTG mRNA by real-time RT-PCR after 24 and 48 h of treatment with 20 µg/ml p31–43 and p57–68. The amount of mRNA of tTG is normalized to that of GAPDH. Values are the means ± SD of three independent experiments. *p<0.05 versus untreated. (**B**) Western blot analysis of tTG protein level after 48 h of treatment with 20 µg/ml p31–43 and p57–68. The blot shown is representative of three independent experiments. (**C**) Densitometric analysis (means of three independent western blot experiments) of protein levels after 24 and 48 h of treatments. The amount of tTG is normalized to that of tubulin. Values are the means ± SD *p<0.05 *versus* untreated.

### RNA Extraction and Real-time RT-PCR

Caco-2 cells were treated with different amounts of p31–43 or p57–68, or alternatively with 1 µM THP for 24 or 48 h. Total RNA was extracted with the Tri-Reagent (Sigma-Aldrich) according to the manufacturer’s instructions. The first-strand cDNA synthesis reaction was performed using the Maxima first-strand cDNA synthesis kit (Fermentas, Milan, Italy) and 1 µg total RNA. The cDNA was then used to amplify human tTG, GRP78, and CHOP transcripts with the following primers: tTG upper, TGCTGTGGAGGAGGGTGACT; tTG lower, ACCAGGCGTTGAAGAGCAAA; GRP78-upper, 5′-CTGGGTACATTTGATCTGACTGG-3′, GRP78-lower, 5′-GCATCCTGGTGGCTTTCCAGCCATTC-3′; CHOP-upper 5′-CTT GGC TGACTG AGG AGG AG-3′, CHOP-lower, 5′-TCA CCA TTC GGT CAATCA GA-3′. The concentration of mRNA was normalized to the concentration of GAPDH, which was obtained with the following primers: upper, 5′-TTCAACAGCGACACCCACTG-3′; lower, 5′- CACCCTGTTGCTGTAGCCA-3′. For PCR reactions cDNA samples were analyzed in triplicate with the iQ™ SYBR Green Supermix (Bio-Rad Laboratories, Milan, Italy) and using the iQ™ 5 Multicolor Real Time PCR Detection System (Bio-Rad Laboratories). PCR reactions were performed with 250 nM of each primer and 10 µl of SYBR Green Supermix, in a total volume of 20 µl. The PCR program started with 3 min of incubation at 95°C, followed by 40 cycles of 15 sec at 95°C, 15 sec at 60°C, and 20 sec at 72°C.

**Figure 7:**
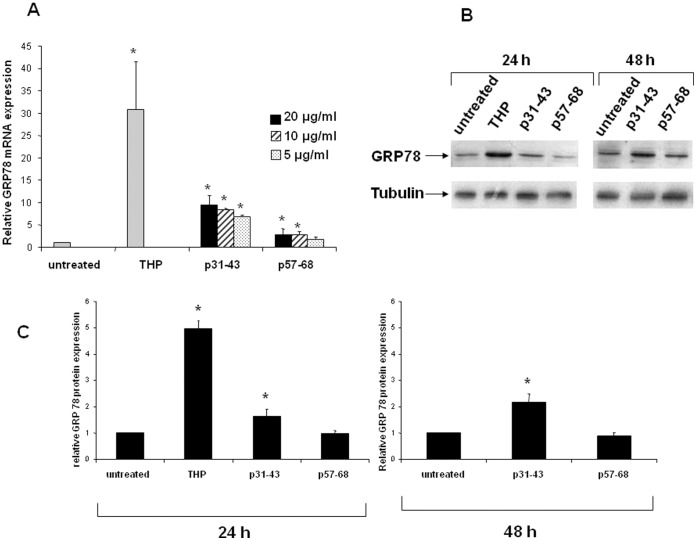
Analysis of GRP78 expression in Caco-2 cells. (**A**) Quantification of GRP78 mRNA by real-time RT-PCR after 24 h of treatment with 1 µM THP, or 20 µg/ml p31–43 and p57–68. The amount of mRNA of GRP78 is normalized to that of GAPDH. Values are the means ± SD of at least three independent experiments. *p<0.05 versus untreated. (**B**) Western blot analysis of GRP78 protein level after 24 and 48 h of treatments with 20 µg/ml p31–43 and p57–68. Cells were exposed to THP (1 µM) for 24 h only. The blot shown is representative of three independent experiments. (**C**) Densitometric analysis (means of three independent western blot experiments). The amount of GRP78 is normalized to that of tubulin. Values are the means ± SD *p<0.05 *versus* untreated.

**Figure 8:**
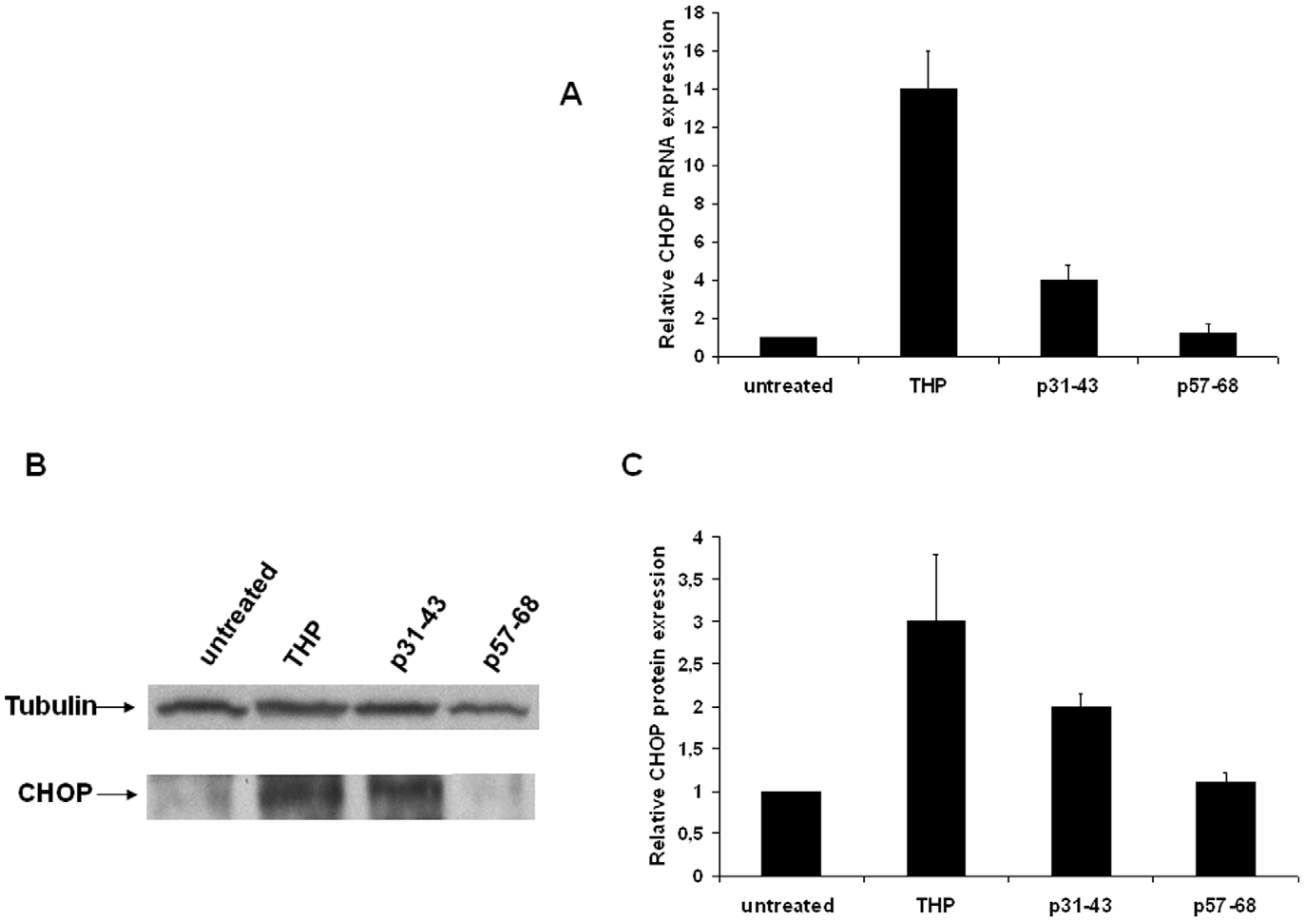
Analysis of CHOP expression in Caco-2 cells. (**A**) Quantification of CHOP mRNA by real-time RT-PCR after 24 h of treatment with 1 µM THP, or 20 µg/ml p31–43 and p57–68. The amount of CHOP mRNA is normalized to that of GAPDH. Values are the means ± SD of three independent experiments. *p<0.05 versus untreated. (**B**) Western blot analysis of CHOP protein level after 24 h of treatments. The blot shown is representative of three independent experiments. (**C**) Densitometric analysis (means of three independent western blot experiments). The amount of CHOP is normalized to that of tubulin. Values are the means ± SD *p<0.05 *versus* untreated.

### Western Blot Analyses

Caco-2 cells were incubated for 24 or 48 h with 20 µg/ml of p31–43 or p57–68 or, alternatively, with 1 µM THP. Cells were washed with phosphate-buffered saline solution and mechanically harvested in lysis buffer, consisting of 20 mM Tris-HCl, pH 7.5, 150 mM NaCl, 1 mM EDTA, 1 mM dithiotreitol, 0.1% sodium dodecyl sulphate, 1% triton X-100, 1 mM ortovanadate, and inhibitors cocktail (Sigma-Aldrich). After 30 min of incubation on ice, cell extracts were centrifuged at 12,000 g for 30 min at 4°C, to remove cell debris, then 75 µg of total proteins were separated by 10% sodium dodecyl sulphate-polyacrylamide gel electrophoresis and transferred to a membrane. To detect tTG, the blot was treated as described elsewhere [23]. To detect GRP78 and CHOP, the blots were treated with 5% skim milk in phosphate-buffered saline overnight at 4°C, then incubated for 2 h with an anti-GRP78 rabbit polyclonal antibody (H-129) (1∶500; Santa Cruz, CA, USA), or with an anti-CHOP mouse monoclonal antibody (1∶1,500; Santa Cruz), respectively, in phosphate-buffered saline containing 0.1% tween-20. After washing, the blots were incubated for 1 h at room temperature with an anti-rabbit-peroxidase secondary antibody (1∶10,000; Bio-Rad Laboratories), or with an anti-mouse-peroxidase secondary antibody (1∶10,000; Bio-Rad Laboratories), respectively. Immunocomplexes were revealed using a chemiluminescence detection kit (Euroclone) according to the manufacturer’s instructions.

### Statistics

Data regarding Ca^2+^ measurements were expressed as mean ± SEM; statistical comparisons were performed using the one-way ANOVA, followed by Newman Keul’s test. Data regarding tTG transamidationg activity and tTG, GRP78, and CHOP expression were expressed as means ± SD; statistical analysis was performed by using the Student’s t test. In all experiments, differences were considered to be statistically significant at p<0.05.

## Results

### Gliadin Peptides 31–43 and 57–68 Increase Intracellular Ca^2+^ Concentrations in Caco-2 Cells

Using single-cell Fura-2 acetoxymethyl ester microfluorimetry, we first investigated whether p31–43 could alter the intracellular Ca^2+^ concentration ([Ca^2+^]_i_) of Caco-2 cells. At 20 µg/ml, p31–43 caused a rapid rise in [Ca^2+^]_i_ when perfused in normal Krebs solution (Figure 1A). Similarly, also p57–68 (20 µg/ml) caused a rapid increase in [Ca^2+^]_i_ when perfused in normal Krebs solution (Figure 1B). The effect of both peptides on [Ca^2+^]_i_ was dose-dependent. However, p31–43 caused a higher [Ca^2+^]_i_ increase than p57–68 (about 65% and 45%, respectively, with respect to the basal value) when tested at 20 µg/ml. At this concentration, pCTR did not affect [Ca^2+^]_i_. Interestingly, also at a low concentration, i.e. 0.2 µg/ml, p31–43 induced an increase in [Ca^2+^]_i_ (45% of basal value), whereas at the same concentration, p57–68 was almost ineffective (Figure 1C and D). Consequently, the EC_50_ for p31–43 and p57–68 was 0.2 µg/ml and 0.9 µg/ml, respectively.

We also evaluated whether p31–43 and p57–68 exert the same effects on [Ca^2+^]_i_ when perfused in a Ca^2+^-free solution. The data reported in Figure 2 show that extracellular Ca^2+^ ions flowing through the plasma membrane did not contribute to the effect of the peptides on [Ca^2+^]_i_. This suggests that the peptide-induced [Ca^2+^]_i_ increase was due to the release of Ca^2+^ from intracellular stores.

### Gliadin Peptides 31–43 and 57–68 mobilize Ca^2+^ from Different Intracellular Ca^2+^ Stores

To identify the intracellular Ca^2+^ stores involved in the mechanism of Ca^2+^ release induced by p31–43 and p57–68, we first used THP to determine whether the ER was involved in the effect of the peptides. Treatment of cells with 1 µM THP significantly reduced the [Ca^2+^]_i_ increase caused by p31–43 (20 µg/ml), but not that caused by p57–68 (20 µg/ml) (Figure 2A, B and C). This finding indicated that the ER was involved in the effect exerted by p31–43, but not by p57–68, on intracellular Ca^2+^ homeostasis. We next investigated whether the residual Ca^2+^ release induced by p31–43 was due to mitochondria, which are an important calcium store within the cell [24]. Using the mitochondrial uncoupler FCCP (300 nM) together with THP, we found that the effect of p31–43 on [Ca^2+^]_i_ was further reduced (Figure 3A and C). In addition, the effect of p57–68 was completely inhibited in the presence of FCCP (Figure 3B and C). These data suggest that p31–43 and p57–68 modulated intracellular Ca^2+^ homeostasis with different mechanisms: p31–43 mobilized Ca^2+^ from the ER and mitochondria, while p57–68 mobilized Ca^2+^ only from mitochondria.

### Gliadin Peptides Activate Intracellular tTG

To investigate whether Ca^2+^ mobilization, induced by gliadin peptides, could activate the crosslinking function of intracellular tTG, we used pentylamine-biotin as tTG substrate to detect and quantify intracellular transamidating activity in Caco-2 cells. We first incubated cells in the presence of 20 µg/ml of p31–43 or p57–68. In both cases, microscopy observation of fixed and permeabilized cells after incubation with FITC-conjugated streptavidin revealed a fluorescent signal, which indicates activation of tTG crosslinking activity (Figure 4A). We obtained a similar fluorescence pattern when we treated cells with ionomycin, a Ca^2+^ ionophore that activates tTG intracellular activity [25]. To verify the specificity of tTG activity, we used cystamin, a well known tTG inhibitor, and found that cystamin inhibited the tTG activation induced by both ionomycin and p31–43 (Figure 4A). On the other hand, fluorescence intensity did not increase in cells treated with pCTR or with the other two irrelevant control peptides (pHTP and p229–246) (Figure 4A). Incubation with 5 µg/ml of p31–43 still produced a good fluorescence signal, whereas incubation with 5 µg/ml of p57–68 produced low fluorescence (not shown). Consequently, at a low concentration, p31–43 was more active than p57–68. We then explored the possibility that gliadin peptides could themselves be *in situ* tTG substrates. To this aim, we treated cells with biotinylated p31–43 or p57–68 for 30 min and we found that, in the presence of ionomycin, both peptides were able to cross-link to intracellular acyl-acceptor substrates (Figure 4B).

After Ca^2+^ mobilization, we observed an increase in intracellular tTG transamidating activity in both the cytosol and nucleus of ionomycin- and peptide-treated cells; this is also evident in the confocal images of cells treated with p31–43 (Figure 4C). Moreover, tTG staining revealed that nuclear tTG, which normally constitutes a significant fraction of total tTG in Caco-2 cells, was often visibly increased in cells after Ca^2+^ mobilization and tTG activation (Figure 4D).

Interestingly, we found that tTG was also activated when we stimulated cells with gliadin peptides in the presence of a Ca^2+^-free medium (i.e., phosphate-buffered saline without Ca^2+^ and Mg^2+^) (Figure 4E). This finding confirms that extracellular Ca^2+^ ions were not involved in tTG activation within the cells and that tTG activation was due to Ca^2+^ mobilization from intracellular stores. Finally, by treating cells with THP, both in the presence and in the absence of extracellular Ca^2+^ ions, we found that Ca^2+^ release from the ER was sufficient to activate intracellular tTG (Figure 4A and E).

To quantify the increase of tTG activity, we carried out a microplate assay on cell homogenates obtained after treatment with p31–43 or p57–68. We found that tTG activity was more than 50% higher in p31–43-treated cells and about 40% higher in p57–68-treated cells than in cells exposed to pentylamine-biotin alone (Figure 5A). Tissue TG activation was similar in THP-treated cells, whereas control peptides had no effect. As expected, we observed a significant reduction of tTG activity, induced by ionomycin or p31–43, when we pretreated cells with cystamin (Figure 5B).

Finally, we evaluated the effect of prolonged treatment with gliadin peptides on tTG expression. As shown in Figure 6, we recorded a moderate increase of tTG mRNA and protein after 24 h of treatment with p31–43 but not with p57–68. However, treatment with p31–43 for 48 h induced a four-fold increase of tTG mRNA expression and a two-fold increase of tTG protein.

### Gliadin Peptide 31–43 Induces GRP78 and CHOP Expression

Since p31–43 can mobilize Ca^2+^ ions from the ER, we asked whether persistent stimulation with p31–43 could induce the ER stress response in Caco-2 cells. To test this hypothesis, we analysed the expression of two well known biochemical marker of ER stress, GRP78 and CHOP ([26], [27].

We treated Caco-2 cells for 24 h with 20 µg/ml of p31–43 or with 1 µM of THP, which is considered an ER stress-inducing agent. We found that, in the presence of THP, GRP78 mRNA expression increased about 30-fold with respect to basal GRP78 expression in untreated cells (Figure 7A). Importantly, in the presence of p31–43, GRP78 mRNA expression increased about nine-fold (Figure 7A), which indicates that p31–43 triggered an ER-stress response in Caco-2 cells. Interestingly, p31–43 induced a very similar GRP78 mRNA increase when used at lower concentrations (i.e., 10 and 5 µg/ml). However, under the afore-mentioned experimental conditions, p57–68 also induced a small but significant increase in GRP78 mRNA expression. We are unable to explain this finding. As shown in Figure 7B and C, the expression of the GRP78 protein increased by about 50% after treatment for 24 h with 20 µg/ml of p31–43. In addition, GRP78 protein expression was doubled after treatment for 48 h with p31–43. Under the same conditions, p57–68 did not induce an increase of GRP78 protein expression. As expected, after treatment with THP for 24 h, GRP78 protein expression was increased about five-fold.

We next measured CHOP expression and found that treatment with THP for 24 h caused an increase of about 14-fold and three-fold in CHOP mRNA and protein expression, respectively (Figure 8). Treatment with p31–43 increased CHOP mRNA expression four-fold, and CHOP protein expression two-fold; p57–68 did not modify CHOP expression (Figure 8).

## Discussion

In CD, the catalytic activity of tTG seems to be crucial for the deamidation of immunogenic gliadin peptides as well as for the formation of gliadin-tTG complexes [1], [4]. Moreover, the catalytic activity of tTG has been associated with an increased inflammatory response to gliadin [18]. However, it is not clear where tTG is activated at cellular and tissue level. Inside cells, tTG is normally latent because of the low Ca^2+^ concentration and the inhibitory effect of GTP/GDP. When activated, tTG can interact with various intracellular proteins and so alter their structure, function, and/or stability [28]–[30]. Interestingly, in a previous proteomic study, we identified many tTG-modified protein targets in human intestinal epithelial cells by using both an acyl-acceptor tTG substrate (pentylamine-biotin) and an acyl-donor tTG substrate (the hexapeptide TVQQEL) [31].

Here we report that treatment of Caco-2 cells with gliadin peptides p31–43 and p57–68 activated intracellular tTG by causing a rapid increase of Ca^2+^ in the cytosol. However, the effect induced by p31–43 was more pronounced than the effect induced by p57–68. Treatment of cells with irrelevant control peptides did not induce tTG activation. Interestingly, as a consequence of tTG activation, p31–43 and p57–68 appear to be cross-linked to cellular acyl-acceptor proteins. This finding is in line with our previous result that tTG mediates p31–43 incorporation into CD mucosal enterocytes *in situ* [23]. The observation that active tTG can recognize and modify gliadin peptides inside the cells led to the notion that also tTG-mediated deamidation could be an intracellular event.

Our finding that the intracellular Ca^2+^ concentration increased in Caco-2 cells also in the absence of extracellular Ca^2+^ ions demonstrates that gliadin peptides induced Ca^2+^ mobilization from intracellular Ca^2+^ deposits. Consequently, we used THP and FCCP, which specifically deplete the ER and mitochondria, respectively, to identify the intracellular Ca^2+^ stores involved in the mechanism of Ca^2+^ release induced by p31–43 and p57–68. Our data reveal two main pathways of Ca^2+^ release, namely, p31–43 modulates intracellular Ca^2+^ homeostasis by mobilizing Ca^2+^ ions from the ER and mitochondria, whereas p57–68 mobilizes Ca^2+^ ions only from mitochondria. Peptides 31–43 and p57–68 can enter cells and localize at vesicular level. In particular, p31–43 localizes in the early vesicular compartment when p57–68 progresses to the late compartment [14], [20]. Peptide 31–43 is delayed in the early compartment because it deranges the correct localization of the hepatocyte growth factor-regulated tyrosine kinase substrate [14]. Indeed, the hepatocyte growth factor-regulated tyrosine kinase substrate is a key regulator of endocytic maturation that acts at the early vesicular compartment. Thus, the different localization of the two gliadin peptides may explain why they mobilized Ca^2+^ ions from different compartments.

We found that tTG was active in the cytosol and very active in the nucleus of Caco-2 cells treated with gliadin peptides, which indicates that tTG translocates into the nucleus in response to the elevated intracellular Ca^2+^ level. The presence of an enhanced level of active tTG enzyme in the nuclear fraction as a consequence of increased tTG import from the cytoplasm to the nucleus is not a novelty [25], [32], [33]. The molecular mechanisms underlying tTG translocation are poorly understood [33]. However, the importance of nuclear tTG in regulating gene expression is well documented. For example, McConoughey et al. [34] reported that, in the nucleus, transamidation can dysregulate the expression of metabolic, chromatin, chaperone and cell death genes in Huntington’s disease. Moreover, Tatsukawa et al. [35] demonstrated that tTG activity is induced in the nuclei of ethanol-treated hepatocytes. Active tTG cross-links and inactivates the general transcription factor Sp1 thereby inducing hepatic apoptosis.

In the cytosol, tTG-catalyzed transamidation of glutamine 63 of RhoA activates RhoA, which plays a key role in the signaling mechanism of the MAPK pathways [36]; it also increases endothelial permeability [37]. In addition, tTG participates in the activation of nuclear factor kappa (NF-κ)B by cross-linking the inhibitory protein IκBα [38]. NF-κB is a transcription factor that is constitutively active in the intestinal mucosa of patients with untreated CD [39] and is considered central to intestinal inflammation [40]–[42]. Finally, it has been hypothesized that tTG-mediated downregulation of the PPARγ signaling pathway in cells treated with p31–43 may contribute to NF-κB activation and therefore plays a pivotal role in CD-associated inflammation [18], [43]. In line with this scenario, we observed that prolonged treatment of Caco-2 cells with p31–43, but not with p57–68, increased tTG mRNA and protein expression in Caco-2 cells. This finding supports the hypothesis that p31–43 could induce tTG-mediated pro-inflammatory modifications in cells.

A large body of evidence suggests that Ca^2+^ is an important regulator of cell fate. An abrupt increase of intracellular Ca^2+^ is found in ischemia-reperfusion injury, receptor over-stimulation and oxidative stress [44]. Depletion of Ca^2+^ from the ER, which is the most important intracellular store of Ca^2+^, can cause protein misfolding and ER-stress. ER-stress triggers a series of signaling and transcriptional events known as the unfolded protein response. The unfolded protein response attempts to restore homeostasis in the ER but, if unsuccessful, can trigger apoptosis in the stressed cells and local inflammation [45]. Chaperone production is upregulated in response to increased misfolding, and the magnitude of the increase in chaperone levels, particularly GRP78, is widely used as a marker of ER-stress [26], [45]. Here we report that stimulation of Caco-2 cells with p31–43 increases GRP78 mRNA expression by about nine-fold, and GRP78 protein levels by about 50% versus basal GRP78 levels in untreated cells. We also evaluated whether prolonged treatment with gliadin peptides could activate CHOP, a transcription factor that primarily mediates stress-linked apoptosis in cells with an irrecoverable level of ER-stress [46]. We found a moderate but significant increase of CHOP expression (both as mRNA and as protein) in cells treated with p31–43, but not with p57–68. Interestingly, it was recently reported that ER-stress-induced CHOP can negatively modulate PPARγ action, thus enhancing the pro-inflammatory response in human intestinal epithelial cells [47].

ER-stress has been associated with an increasing number and wide variety of human diseases, namely, cancer, diabetes, developmental disorders, and neurodegenerative, infectious and inflammatory diseases [48]. The concept that ER-stress, elicited by the gliadin peptide p31–43, could be involved in CD is intriguing also because unresolved ER-stress leads to intestinal epithelial cell dysfunctions typical of the entity inflammatory bowel disease [49].

**Figure 9:**
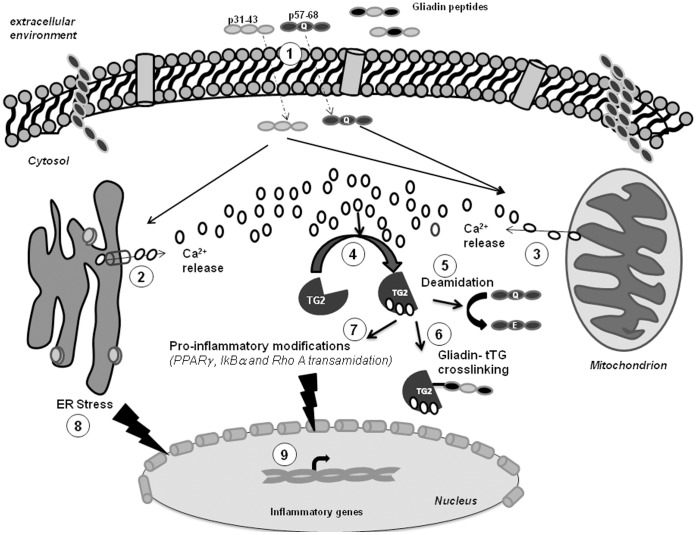
A model of the possible relationship between gliadin peptides, tTG activation, ER-stress and the inflammatory response. Toxic and immunogenic gliadin peptides penetrate inside the cells through vesicular trafficking (1) [14], [18], [20] and rapidly induce Ca^2+^ release from the ER (2) and mitochondria (3). The increased [Ca^2+^]_i_ activates normally silent tTG (4), which in turn deamidates gliadin peptides (5) and/or produces cross-links between the peptides and the tTG itself (6) or between the peptides and other cellular proteins [4], [5], [11], [12]. In addition, active tTG transamidates IκBα [38], PPARγ [18], and Rho A [36], which are key regulators of the inflammatory response. Persistent stimulation with toxic gliadin peptides (p31–43) can also trigger an ER-stress response (8) that, in turn, can modulate the expression of inflammatory genes (9) [45].

In conclusion, given the direct links between ER-stress/unfolded protein response and local and systemic inflammation, we suggest that p31–43, which is responsible for innate immunity in CD, could promote an ER-stress pathway by inducing rapid Ca^2+^ mobilization from the ER, and so amplify a local inflammatory response (Figure 9). Moreover, by mobilizing Ca^2+^ from intracellular stores, both p31–43 and p57–68 could induce tTG-mediated modifications of several key regulators of the inflammatory response. Finally, intracellular tTG activation could allow deamidation of immunogenic gliadin peptides and the formation of gliadin-tTG complexes (Figure 9) inside enterocytes and other specialized antigen-presenting cells, such as duodenal dendritic cells and macrophages, which contain large amounts of tTG.
